# Pre-existing diabetes mellitus and adverse pregnancy outcomes

**DOI:** 10.1186/1756-0500-5-496

**Published:** 2012-09-10

**Authors:** Hayfaa A Wahabi, Samia A Esmaeil, Amel Fayed, Ghadeer Al-Shaikh, Rasmieh A Alzeidan

**Affiliations:** 1 Sheikh Bahmdan Chair of Evidence-Based Healthcare and Knowledge Translation, College of Medicine, King Saud University, Riyadh, Kingdom of Saudi Arabia; 2 King Saud Ben AbdulAziz University for Health Sciences, Riyadh, Kingdom of Saudi Arabia; 3 High Institute of Public Health Alexandria University, Alexandria, Egypt; 4 Obstetrics and Gynecology Department, College of Medicine, King Saud University, King Khalid University Hospital, Riyadh, Kingdom of Saudi Arabia

**Keywords:** Pre-existing diabetes mellitus, Macrosomia, Stillbirth, Cesarean delivery, Preterm delivery

## Abstract

**Background:**

Pregnancies complicated by pre-existing diabetes mellitus (PDM) are associated with a high rate of adverse outcomes, including an increased miscarriage rate, preterm delivery, preeclampsia, perinatal mortality and congenital malformations; compared to the background population. The objectives of this study are to determine the prevalence of PDM and to investigate the maternal and the neonatal outcomes of women with PDM.

**Methods:**

This is a retrospective cohort study for women who delivered in King Khalid University Hospital (KKUH) during the period of January 1^st^ to the 31^st^ of December 2008. The pregnancy outcomes of the women with PDM were compared to the outcomes of all non-diabetic women who delivered during the same study period.

**Results:**

A total of 3157 deliveries met the inclusion criteria. Out of the study population 116 (3.7%) women had PDM. There were 66 (57%) women with type 1 diabetes mellitus (T1DM) and 50 (43%) women with type 2 diabetes mellitus (T2DM). Compared to non-diabetic women those with PDM were significantly older, of higher parity, and they had more previous miscarriages. Women with PDM were more likely to be delivered by emergency cesarean section (C/S), OR 2.67, 95% confidence intervals (CI) (1.63-4.32), *P* < 0.001, or elective C/S, OR 6.73, 95% CI (3.99-11.31), *P* < 0.001. The neonates of the mothers with PDM were significantly heavier, *P* < 0.001; and more frequently macrosomic; OR 3.97, 95% CI (2.03-7.65), *P* = 0.002. They more frequently have APGAR scores <7 in 5 minutes, OR 2.61, 95% CI (0.89-7.05), *P* 0.057 and more likely to be delivered at <37 gestation weeks, OR 2.24, 95% CI (1.37- 3.67), *P* 0.003. The stillbirth rate was 2.6 times more among the women with PDM; however the difference did not reach statistical significance, *P* 0.084.

**Conclusion:**

PDM is associated with increased risk for C/S delivery, macrosomia, stillbirth, preterm delivery and low APGAR scores at 5 min.

## Background

It is estimated that by the year 2030 more than 360 million people will have diabetes mellitus (DM) [1] and as the burden of the disease increases the management of pregnancies complicated by DM will be part of the daily obstetric practice in many regions of the world. Studies investigating the influence of ethnicity on the outcome of pregnancies complicated by pre-existing diabetes mellitus (PDM) reported variation in the outcome with different ethnic groups with worse outcome for Asian [2] and Afro-Caribbean mothers compared to Caucasian [3], however this difference might be explained by access to and utilization of preconception and prenatal care [3].

The physiological changes of pregnancy put the human body in a state of carbohydrate intolerance. The pregnancy specific hormones, such as human placental lactogen and the increased levels of cortisol and prolactin, increase the resistance to insulin and call for more production of the hormone to maintain homeostasis of blood glucose during pregnancy [4]. Such demand is not met in pregnant diabetic women due to the pathology associated with diabetes.

Pregnancies complicated by diabetes are associated with a high rate of miscarriage, preterm delivery, preeclampsia, perinatal mortality and congenital malformations compared to the background population [5,6]. A recent systematic review showed that pregnancies complicated by type 2 diabetes mellitus (T2DM) are associated with worse perinatal mortality and neonatal mortality than those complicated by type 1 diabetes mellitus (T1DM) [7].

Many diabetes associated complications such as congenital malformations are due to the effect of maternal hyperglycemia on the developing embryo during the early weeks of conception [8], thus preconception care including normalization of blood glucose, folic acid supplementation and detection and treatment of diabetic vascular complications, has significantly improved the rate of congenital malformations, perinatal mortality and preterm delivery [9]. Close monitoring and normalization of blood glucose for pregnant women with T1DM and T2DM significantly improve the perinatal mortality and the stillbirth rate [10].

The objectives of this study are to determine the prevalence of PDM and to investigate the maternal and the neonatal outcomes of women with PDM.

## Methods

This is a retrospective cohort study to investigate the maternal and the neonatal outcomes of diabetic women with PDM (type 1 and type 2) who were admitted to the labour ward in King Khalid University Hospital (KKUH) for delivery as compared to the non-diabetic women delivered during the same period.

In KKUH maternity unit, women with PDM follow a specific course of treatment including nutritional therapy, self-monitoring of blood glucose level and antenatal fetal surveillance. All women with T2DM, who used oral hypoglycemic agents when not pregnant, are shifted to insulin during pregnancy. Insulin therapy is adjusted to maintain the fasting and pre-prandial blood glucose at ≤5.9 mmol/l (106 mg/100 ml) and the 1 h postprandial glucose at < 7.8 mmol/l (140 mg/dl) [11].

The data for this study were collected for the period of 12 months from the 1^st^ of January to the 31^st^ of December 2008 from the labour ward registry and the missing data were obtained from the maternal medical records. The inclusion criteria for this study were:

1. Gestation age of 24 weeks or more at the time of delivery, calculated from the last menstrual period and/or early ultrasound scan.

2. Singleton pregnancy.

3. Women diagnosed with either T1MD or T2DM before the index pregnancy (study group).

4. Women with neither PDM nor gestational diabetes (GDM) (control group).

The demographic characteristics and the pregnancy outcomes of the women with PDM were compared to the outcomes of all non-diabetic women who delivered during the same study period. Women with multiple pregnancies and those diagnosed with GDM were excluded from the analysis of the outcomes; however all deliveries more than 24 weeks were considered when calculating the prevalence rate of PDM. Further analysis was done to compare the outcomes of pregnancies complicated with T1DM and T2DM. The maternal variables we assessed were; age, gravidity, parity, gestation age at delivery, mode of delivery, premature delivery at less than 37 weeks of gestation and previous history of miscarriage. The neonatal outcomes included birth weight, macrosomia (birth weight ≥ 4 kg) and APGAR scores at 5 min after delivery and the prevalence of intra-uterine fetal death (IUFD).

### Statistic analysis

We compared means using the Student *t* test for continuous variables after assessing normality distribution of the variables, and *Chi squire* for categorical variables. Data were analyzed using Statistical Package for the Social Sciences (SPSS), Version 17 (SPSS Inc., Chicago, IL, USA). Odds Ratio (OR) was calculated and *P*-value of less than 0.05 was considered significant. Outcomes for macrosomia and mode of delivery were adjusted for maternal age and parity using regression analysis.

Ethical approval was sought and granted before commencing the study from the institutional ethics review board of King Saud University Medical College.

## Results

There were 3273 deliveries during the study period of which 3157 met the inclusion criteria. Of the study population 116 (3.7%) women had PDM and 569 had GDM (18.0%) while 2472 (78.3%) were not diabetic. For outcomes analysis, the 2472 non-diabetic women were compared to the 116 women in the PDM group.

There were 66 (57%) women with T1DM and 50 (43%) women with T2DM. All women with PDM received insulin during pregnancy to reach the target blood glucose levels.

The comparisons of the demographic characteristics of the women with PDM and non-diabetic women are shown on ` 1. The diabetic mothers were significantly older; they had significantly more pregnancies and of significantly higher parity, in addition significantly more of the diabetic women had previous miscarriages compared to the non-diabetic women. Compared to the non-diabetic group the women with PDM were nearly threefold more likely to be delivered by emergency cesarean section (C/S), OR 2.67, 95% confidence intervals (CI) (1.63-4.32), P < 0.001, and more than six fold more likely to deliver by elective C/S, OR 6.73, 95% CI (3.99-11.31), *P* < 0.001 (Table 2). The neonates of the mothers with PDM were significantly heavier when compared to those of the non-diabetic mothers, *P* < 0.001; and the odds for macrosomia were increased to almost fourfold for the neonates of the PDM group OR 3.97, 95% CI (2.14-7.40), *P* < 0.001. The mean gestational age at delivery for mothers with PDM was significantly less than the non-diabetic mothers 38.67 ± 2.295 versus 37.28 ± 2.812, *P* < 0.001. There was twofold increase in preterm delivery less than 37 gestation weeks, OR 2.24, 95% CI (1.37- 3.67), *P =* 0.003 (Table 2). Although there was a threefold increase in the odds for low APGAR scores among the neonates of the mothers with PDM compared to non diabetics, OR 2.61, 95% CI (0.89- 7.05); the difference did not reach statistical significance, *P =* 0.057. Similarly, despite the increase in frequency of intra-uterine fetal death (IUFD) among women with PDM compared to non-diabetic women, the difference did not reach statistical significance, *P =* 0.084; however the OR of 2.62, 95% CI (0.78-7.96) was consistent with more than twofold increase (Table 2).

**Table 1:** Comparison of maternal demographic characteristics between non-diabetic women, and women with pre-existing diabetes mellitus

**Characteristic**	**Non-diabetic 2472**	**Pre-existing Diabetes Mellitus116**	**P value**
Maternal age	28.62 ± 5.98	34.95 ± 5.66	< 0.001
Gravidity	3.58 ± 2.78	6.43 ± 3.46	< 0.001
Parity	2.06 ± 2.28	4.53 ± 2.90	< 0.001
History of previous miscarriage	786 (31.8)	54 (46.6)	0.002

Data expressed as means ± standard deviation or n (%).

**Table 2:** Comparison of maternal and perinatal outcomes between non-diabetic women, and women with pre-existing diabetes mellitus

**Characteristic**	**Non-diabetic 2472 (95.5)**	**Pre-existing Diabetes Mellitus 116 (4.5)**	**OR (95%CI)**	**P value**
Emergency C/S	340 (13.8)	28 (24.1)	2.67(1.64-4.32)	< 0.001
Elective C/S	125 (5.1)	26 (22.4)	6.73(3.99-11.31)	< 0.001
APGAR scores at 5 min <7	42 (1.7)	5 (4.3)	2.61(0.89-7.05)	0. 057
Birth weight	3120.14 ±578.18	3227.34 ± 804.88		< 0.001
Macrsomia	76 (3.1)	13 (11.2)	3.97 (2.14-7.40)	< 0.001
IUFD	32 (1.3)	4 (3.4)	2.62 (0.78-7.96)	0.084
Mean gestation age at delivery (weeks)	38.67 ± 2.295	37.28 ± 2.812		<0.001
Delivery < 37 weeks	222 (9)	21(18.1)	2.24 (1.37- 3.67)	0.003

OR = Odd Ratio, CI = Confidence intervals, C/S = Cesarean section, IUFD = Intra-uterine fetal death. Data expressed as means +/- standard deviation or number (%).

After adjustment for maternal age and parity, women with PDM continued to show significantly higher odds for C/S delivery OR =3.5, 95% CI; (2.34-5.24), *P* <0.001 and macrosomia OR = 2.71, 95% CI; (1.41-5.21), *P* <0.001 than non-diabetic women.

There were no significant differences in the outcomes between pregnancies complicated by T1DM and T2DM except for the previous miscarriage which was significantly more frequent among women with T2DM; *P =* 0.015 (Table 3).

**Table 3:** Maternal characteristic and outcomes of pregnancy complicated with type 1 and type 2 diabetes mellitus

**Pregnancy outcomes**	**T1DM n = 50**	**T2DM n = 66**	**P value**
Maternal age (years)	34.44 ± 6.142	35.33 ± 5.298	0.403
Parity	4.04 ± 3.221	4.89 ± 2.603	0.119
Birth weight (gms)	3223.08 ± 856.193	3230.58 ± 770.400	0.961
Previous miscarriage	17(14.7)	37(31.9)	0.015
Cesarean section delivery	26 (22.4)	28 (24.1)	0.350
APGAR scores at 5 min <7	3 (2.6)	2 (1.7)	0. 651
Macrsomia	6 (5.2)	7 (6.0)	1.0
IUFD	2 (1.7)	2 (1.7)	1.0
Delivery < 37 weeks	9 (7.8)	12(10.3)	1.0

Data presented as mean ± standard deviation or number and percentage, IUFD = Intrauterine fetal death.

## Discussion

The prevalence of PDM in this study is 3.7% which indicates a fivefold increase during the last 14 years based on earlier studies from Saudi Arabia [12,13]. This noticeable rise in the prevalence of PDM during pregnancy could be explained in part by the improved screening and detection of diabetes, however the main cause in our opinion is the increased prevalence of T2DM among adults in the Saudi population to an estimated range of 21to 23% [14]. It is worth noting that the prevalence of 3.7% for PDM is very high compared to other regions of the world [6,15-17].

Our results confirm the findings by other investigators about the demographic characteristics of the diabetic pregnant women. In this study women with PDM are older and of higher parity when compared to non-diabetic pregnant women [12]. Moreover diabetic women have poorer reproductive performance including an increased rate of miscarriage which concurred with the findings in similar cohort [18,19].

We observed a threefold increase in the rate of APGAR scores less than 7 at 5 min of birth on the diabetic women compared to the non-diabetic. In a recent report low APGAR scores are predictors of long term neurological disabilities [20]. Such low scores in the diabetic cohort could be explained by the increase risk of birth asphyxia due to placental angiopathy [21].

Our results indicated that infants of diabetic mothers had significantly higher birth weight compared to those of non-diabetic mothers; moreover 11% of the infants delivered to the diabetic mothers were macrosomic compared to 3% of those delivered to non-diabetic mothers (Table 2). Uncontrolled maternal hyperglycemia adversely influences fetal weight and growth with resultant macrosomia at moderately elevated levels and intra-uterine growth restriction at very high levels of maternal blood glucose [21]; however PDM is not the only factor which causes fetal macrosomia other factors including maternal age over 30 years, prolonged pregnancy, multiparity [22] and maternal obesity were proven to play a role in controlling birth weight [23]. Macrosomia is associated with significant maternal and perinatal complications including increased rate of C/S, birth asphyxia and perinatal mortality [24].

The results of this study showed that almost 50% of women with PDM had C/S delivery; while the corresponding figure for non-diabetic women was less than 20%. This finding agrees with that observed by other investigators [12,15]. The reason behind the tendency towards delivery by C/S is in great part attributed to the increased rate of macrosomia among women with PDM, however significant association was found between the risk of C/S delivery in diabetic women and maternal obesity, uncontrolled diabetes and unplanned pregnancy [25]. Recent reports found that with the increase rate of elective C/S there was improvement in the rate of shoulder dystocia and its associated morbidities [26] as well as APGAR scores at 5 min [27], nevertheless the effectiveness and cost effectiveness of the approach of screening for macrosomia by ultrasound scanning, fetal weight estimation and subsequent delivery by elective C/S was doubted by other investigators [28].

Concurrent with our results is a recent report which confirmed that the rate of both iatrogenic and spontaneous preterm deliveries are increased in mothers who are diabetic, compared to the background population [29] nevertheless, premature infants of diabetic mothers do not seem to be at risk of complications more than the preterm infants of non-diabetic mothers [30].

The stillbirth rate among the women with PDM was almost threefold increased compared to the non-diabetic cohort (Table 2). Recent review on the causes of perinatal mortality in women with PDM showed that antepartum asphyxia and congenital abnormalities were the leading two causes of stillbirth [21]. Placental angiopathy secondary to uncontrolled maternal hyperglycemia was suggested as an etiology for antenatal asphyxia [21] and peri-conception uncontrolled hyperglycemia as the cause of congenital abnormalities [9].

Contrary to the findings by Murphy et al. [31], we did not find significant differences in the maternal characteristics or the outcomes of pregnancies complicated by T1DM compared to those complicated by T2DM except for the history of previous miscarriage (Table 3). This might be due to the small number of women with T1DM and T2DM in this study.

The importance of this study is that it investigated a key public health problem which is PDM and that it gives preliminary indicators about its prevalence and impact on pregnancy outcome in the Kingdom of Saudi Arabia, such information is imperative for practice and research considering the paucity of data about the reproductive outcome of diabetic pregnancy.

We are aware of the limitation of this study including the retrospective nature of the design and the lack of data on important confounding factors which could have influenced the outcome such as maternal pre-pregnancy weight and weight gain during pregnancy, as well as important outcomes such as preeclampsia, congenital malformations and perinatal mortality. Other limitations include a single centre experience which might hinder the extrapolation of the results to other regions of the Kingdom, however, our results are consistent with the national epidemiological studies on T2DM in the Kingdom.

### The implication to practice

Because of the documented high prevalence of T2DM in the Saudi population we recommend that all pregnant women be screened early in pregnancy (during the first trimester) using fasting blood glucose to identify women with PDM [32]. Close monitoring and adjustment of insulin therapy based on daily self-monitoring of blood glucose with clear target of blood glucose level values for fasting and postprandial and periodically assessed hemoglobin A1C levels, is imperative for improving the outcomes for women with PDM [33].

### Implication to research

The results of this study showed that 3.7% of women giving birth every year in KKUH have PDM and an estimated 18% develop GDM, all these mothers and their offspring are at risk of adverse pregnancy outcomes. This high prevalence in one health institution calls for a national prospective survey to estimate the prevalence of PDM in the different regions of the Kingdom and to evaluate the national impact of the disease on the maternal and perinatal outcomes. Such a survey will provide important information for health policy and reproductive health services provision.

## Conclusion

The prevalence of PDM in KKUH is among the highest in the world. PDM is associated with increased risk for C/S delivery, macrosomia, stillbirth, preterm delivery and low APGAR scores at 5 min.

## Competing interest

The authors of this article declare that they have no conflict of interest.

## Authors’ contribution

HW conceived the idea for the study; she supervised the data collection and analysis and she wrote the final manuscript. SE managed the data. AF analyzed the data and extracted the results. RA participated in the analysis. GA supervised data collection and management. All authors read the final draft of the manuscript and approved it.
